# Sensing study of quinoxaline analogues with theoretical calculation, single-crystal X-ray structure and real application in commercial fruit juices

**DOI:** 10.1098/rsos.180149

**Published:** 2018-06-06

**Authors:** Shampa Chakraborty, Shyamaprosad Goswami, Ching Kheng Quah, Bholanath Pakhira

**Affiliations:** 1 Department of Chemistry, Indian Institute of Engineering Science and Technology, Shibpur, Howrah, West Bengal 711103, India; 2 X-ray Crystallography Unit, School of Physics, Universiti Sains Malaysia, 11800 USM, Penang, Malaysia

**Keywords:** chemosensors, nickel, iron(iii), crystal

## Abstract

Single-crystal X-ray structures of dimeric quinoxaline aldehyde (QA), quinoxaline dihydrazone (DHQ) and HQNM (Goswami S *et al.* 2013 *Tetrahedron Lett.* **54**, 5075–5077. (doi:10.1016/j.tetlet.2013.07.051); Goswami S *et al.* 2014 *RSC Adv.* **4**, 20 922–20 926. (doi:10.1039/C4RA00594E); Goswami S *et al.* 2014 *New J. Chem.* **38**, 6230–6235. (doi:10.1039/C4NJ01498G)) are reported along with the theoretical study. Among them, QA is not acting as an active probe, but DHQ and HQNM are serving as selective and sensitive probe for the Fe^3+^ cation and the Ni^2+^ cation, respectively. DHQ can also detect the Fe^3+^ in commercial fruit juices (grape and pomegranate).

## 1. Introduction

The design of a colorimetric cation sensor is important and useful because the colorimetric sensing system would allow ‘naked-eye’ detection of cations without the use of any spectroscopic instrumentation, being simple and convenient for detection. In particular, ratiometric sensors have the important feature of permitting signal rationing, and increase the dynamic range and provide built-in correction for the environmental effect. Such colorimetric/ratiometric receptors would be more valuable if they can be obtained by a simple synthetic method. The important biological activities of quinoxaline derivatives [1–7] include anti-cancer, [8] antimicrobial/anti-tubercular [9], anti-protozoal [10], antiviral [11], inhibition of the enzyme phosphodiesterase [12] anti-inflammatory [13], anti-oxidant [13], anti-tumour and anti-hyperglycaemic activity, etc. Quinoxaline derivatives are known for their cancer chemopreventive effect [8]. Furthermore, the quinoxaline ring is a core structure of several drug molecules and acceptors such as clofazimine, echinomycin and actinomycin [14–17]. Recently, we have also reported some quinoxaline-based colorimetric and ratiometric sensors for specific detection of nickel cations [18–20]. Nickel is a toxic metal and known to cause pneumonitis, asthma and cancer of the lungs, and also disorders of the respiratory and the central nervous system [17,21–24]. Nickel is an essential trace element in biological systems with relevance to the biosynthesis and metabolism in certain microorganisms and plants. Nickel is used in various industries, e.g. in Ni–Cd batteries, rods for arc welding, pigments for paints, ceramics, electroplating, dental and surgical prostheses, and catalysts for hydrogenation, and as magnetic tapes of computers. On the other hand, iron is one of the essential elements for fulfilling physiological function in the human body. Iron plays key roles in various important biological processes at the cellular level, ranging from electron transfer, cellular metabolism, energy generation, gene expression, neurotransmission, regulation of metalloenzymes, DNA synthesis as well as differentiation [25,26]. In particular, its deficiency or overload can cause various disorders and diseases such as anaemia and haemochromatosis. Thus, the development of the sensitive and selective detection approaches of Fe^3+^ in biological systems is of great importance for investigating the physiological and pathological functions of Fe^3+^ in living organisms. In this paper, we investigate the behaviour of three quinoxaline-based derivatives where they act as an active sensor for metal cations and also its real-world application in detection of iron in fruit juices.

## 2. Results and discussion

The design and synthesis of sensors for the detection of a selective metal ion in an aqueous or non-aqueous medium is an active area of research today. Colorimetric sensors are important due to their simplicity and lower capital cost compared with the other closely related methods. Accordingly, the development of a novel colorimetric chemosensor for the rapid and convenient detection of Ni^2+^ and Fe^3+^ is attractive. The binding behaviour of receptor (HQNM) [18] has already been established, hence, in this article, we are discussing more about DHQ and its sensing behaviour with different cations.

**Scheme 1.**
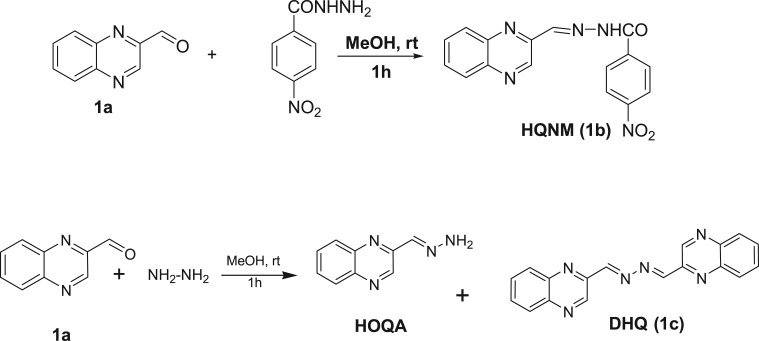
Brief synthesis of the HQNM (1b) and DHQ (1c) from QA (1a).

**Scheme 2.**
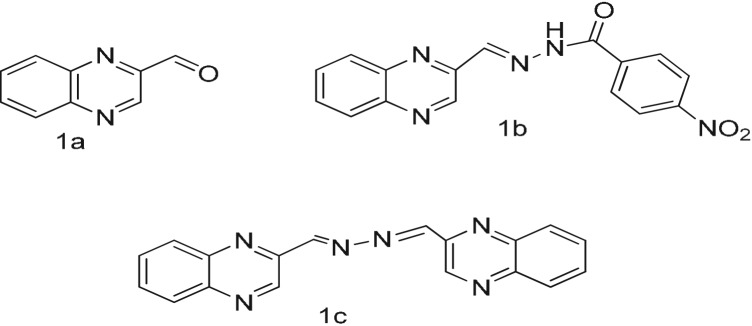
Chemical structures of (1a) QA (1b) HQNM (1c) Quinoxaline DHQ.

The titration was carried out in CH_3_CN-HEPES buffer (9 : 1, v/v, pH = 7.4) at a 1 × 10^−5^ M concentration of HQNM and DHQ upon addition of incremental amounts of 0–200 µl of nickel chloride solution (2 × 10^−4^ M) and ferric chloride solution (2 × 10^−4^ M), respectively (schemes 1 and 2).

**Figure 1.**
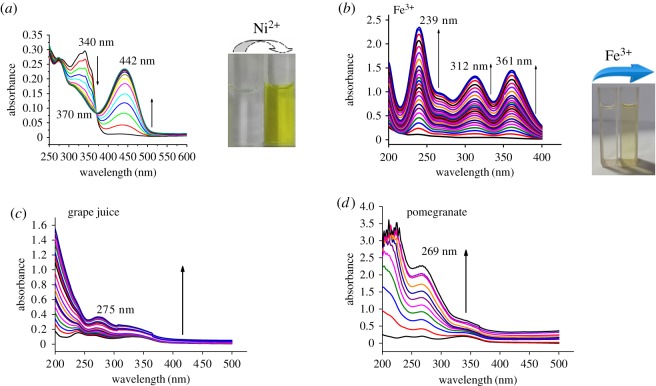
(*a*) UV–vis absorption spectra of HQNM (1 × 10^−5^ M) in CH_3_CN-HEPES buffer (9 : 1, v/v, pH = 7.4) upon titration with nickel chloride (NiCl_2_.6H_2_O, 0.8 equivalent). The arrows show changes due to the increasing concentration of Ni^2+^. Inset: colour change due to the addition of nickel chloride. (*b*) UV–vis absorption spectra of DHQ (1 × 10^−5^ M) in CH_3_CN-HEPES buffer (9 : 1, v/v, pH = 7.4) upon titration with ferric chloride (FeCl_3_.6H_2_O, 0.8 equivalent). The arrows show changes due to the increasing concentration of Fe^3+^. Inset: colour change due to the addition of ferric chloride. (*c*) UV–vis absorption spectra of DHQ (1 × 10^−5^ M) in CH_3_CN-HEPES buffer (9 : 1, v/v, pH = 7.4) upon titration with commercial grape juice. The arrows show changes due to the increasing concentration of Fe^3+^. (*d*) UV–vis absorption spectra of DHQ (1 × 10^−5^ M) in CH_3_CN-HEPES buffer (9 : 1, v/v, pH = 7.4) upon titration with commercial pomegranate juice. The arrows show changes due to the increasing concentration of Fe^3+^.

The UV-visible spectrum of the receptor HQNM [18] with Ni^2+^ is characterized by two bands centred at 340 and 442 nm (figure 1). As shown in figure 1*a*, upon gradually increasing the nickel ion concentration, the band at 340 nm gradually weakens and a new band appears at 442 nm with an isosbestic point at 370 nm. The UV-visible spectrum of the DHQ and Fe^3+^ is shown in figure 1*b*; it was also characterized by three bands centred at 239, 312 and 361 nm. Upon gradually increasing the iron ion concentration, a gradual increase of each band in the UV-visible spectrum is seen. The UV-visible spectrum of DHQ with commercial grape and pomegranate juices is also shown in figure 1*c* and 1*d,* respectively. In figure 1*c*, upon gradually increasing the grape juice concentration (20 µl), the band at 275 nm gradually increases. Similarly in figure 1*d*, upon gradually increasing the pomegranate juice concentration (20 µl), the band at 275 nm gradually increases. Thus the UV–vis absorption spectra of Fe^3+^ in fruit juices could be detected and estimated [27–30] in an aqueous medium and in commercial fruit juice. A control experiment without receptor and only CH_3_CN-HEPES buffer (9 : 1, v/v, pH = 7.4) with fruit juices has been carried out without any significant enhancement of absorbance (electronic supplementary material, figure S9a and S9b).

**Figure 2.**
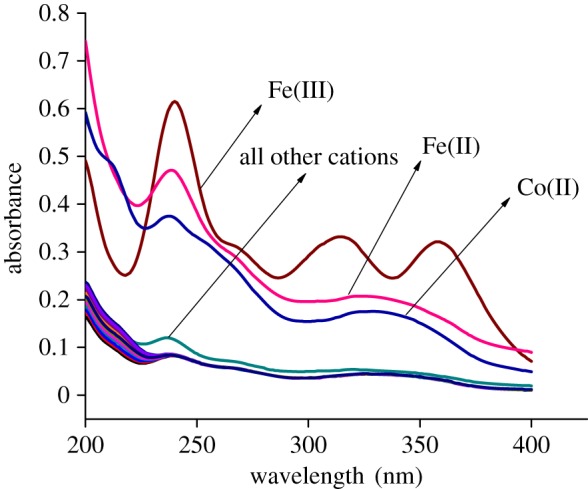
The absorption spectra of DHQ (1 × 10^−5^ M) and DHQ with all other cations (2 × 10^−4^ M) in acetonitrile–HEPES buffer (9 : 1, v/v, pH = 7.4).

The selectivity for the ferric ion over the other cations is shown by plotting the UV–vis spectra diagram of DHQ with different cations. In figure 2, the selectivity for Fe^3+^ is shown by the brown spectrum. However, when titration of other cations such as Ni^2+^, Cu^2+^, Cd^2+^, Zn^2+^, Na^+^, K^+^, Mn^2+^, Hg^2+^ was performed in similar experimental conditions, no significant change in the spectrum for most of the cations was noted except for Co^2+^ and Fe^2+^, but the colour change for those metal cations is not detectable with the naked eye.

From the experimental data, it can be concluded that the receptor DHQ possesses high selectivity and sensitivity towards the iron (III) cation in acetonitrile–HEPES buffer (9 : 1, v/v, pH = 7.4) medium. The other cations had no practical significant influence. The colour changes are most probably due to the formation of coordinate bonds of receptor DHQ on the addition of the ferric ion, which is shown in figure 1*b* (inset).

**Scheme 3.**
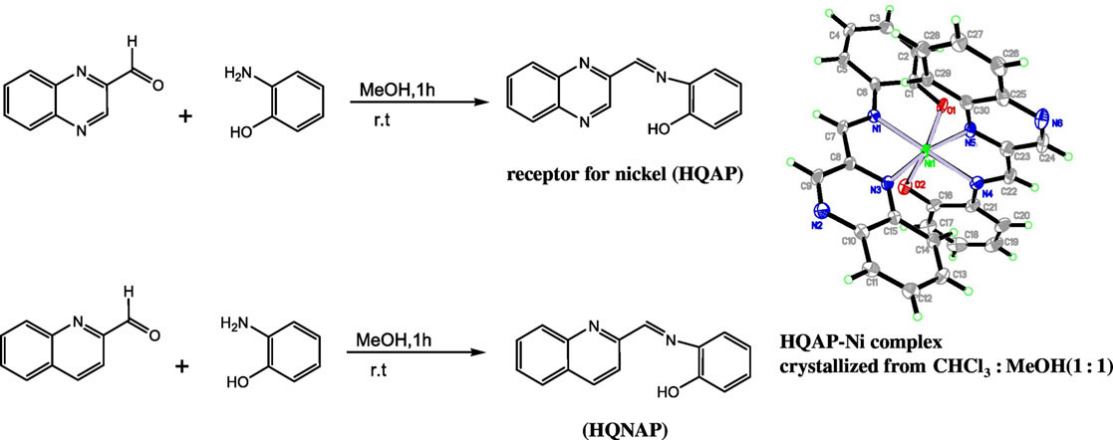
Synthesis of the receptor (HQAP) and HQNAP.

To further explore the binding mechanism, Job's plot of the UV–vis titrations of the Fe^3+^ ion with a total volume of 2 ml was revealed. Maximum absorption was observed when the molar fraction reached 0.65, which is indicative of a 2 : 1 stoichiometric complexation between DHQ and the Fe^3+^ ion for the newly formed species. The electrospray ionization (ESI) mass spectrum of a mixture of DHQ and FeCl_3_.6H_2_O also revealed the formation of a 2 : 1 ligand–metal complex through the metal coordination interaction, with a major signal at *m/z* = 679.0 (possibly for (2M + Fe)^+^ ions) (scheme 3).

**Scheme 4.**
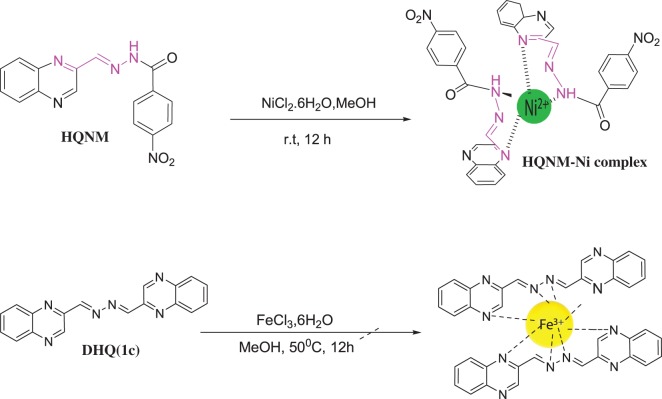
Probable host–guest binding of HQNM and DHQ in the solution phase [18].

The binding selectivity for HQNM (scheme 2 and figure 1*a*) is greatly influenced based on charge–charge interactions, and the involvement of both N–H … Ni bonds, which are absent in the case of quinoxaline aldehyde (QA) and DHQ, as they already exist as dimers, as seen from their crystal structure (figure 2). In accordance with this discussion, for a similar type of compound acting as an active probe or not, two more examples from our previous work are HQNAP (quinoline-2-ylmethyleneamine, scheme 2) and HQAP^1b^(quinoxalin-2-ylmethyleneamine). In contrast to our previous receptor HQAP [18], HQNAP does not serve as a good nickel sensor (electronic supplementary material, figure S8). Surprisingly, from the complex crystal structure of HQAP, we cannot see any direct bond with the second quinoxaline nitrogen to the nickel. However, from that fact we can say that there must be some effect of the second quinoxaline nitrogen on the HQAP–Ni complex so that it will be formed, and of the quinoline moiety, so that it will not form at all (scheme 4).

### 2.1. X-ray crystallography

**Figure 3.**
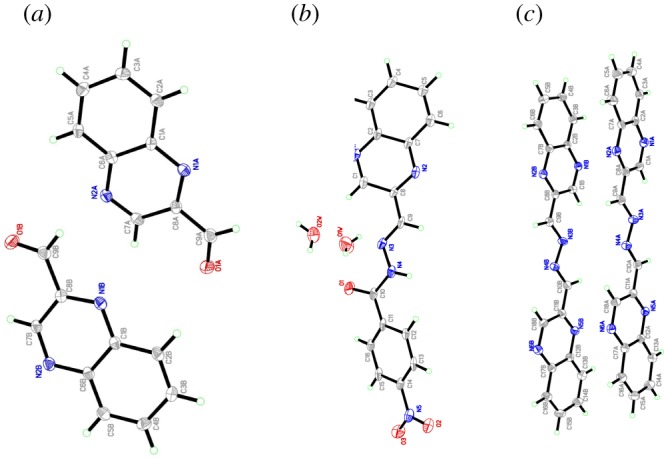
The molecular structures of (*a*) QA, (*b*) HQNM and (*c*) DHQ, showing 50% probability displacement ellipsoids for non-H atoms and the atom-numbering scheme.

The X-ray structure of QA has been reported with a CCDC number CCDC 978283. For QA, the asymmetric unit consists of two molecules, with comparable geometries (figure 3*a*) which are approximately planar (for the 12 non-H atoms). There are no significant hydrogen bonds observed in the crystal structure, and molecules are stacked along the axis (electronic supplementary material, figure S5) by way of weak aromatic π–π stacking interactions between the benzene rings in adjacent molecules. The X-ray structure of HQNM has been reported with a CCDC number CCDC 1023223.

The compound HQNM (figure 3*b*) consists of a HQNM molecule and 1.5 water molecule in the asymmetric unit, and exists in trans conformations related to the N3=C9 bond. One of the water molecules lies in a mirror plane. The dihedral angle between the two benzene rings is 38.3 (4)°. In the crystal, molecules are linked into two-dimensional planes (electronic supplementary material, figure S6) lying parallel to (10-1) via intermolecular C—H ··· O hydrogen bonds (electronic supplementary material, table S2). Adjacent planes are cross-linked via water molecules with further O–H ··· O, N–H ··· O and C–H ··· O interactions into a three-dimensional network (electronic supplementary material, figure S6c). The crystal packing is further consolidated by π–π stacking interactions between the two symmetry-related benzene rings.

The asymmetric unit of DHQ (figure 3*c*) contains two crystallographically independent molecules, both of which exist in trans, trans conformations related to the N3=C9 and N4=C10 bonds. The non-H atoms of the monohydrazone quinoxaline moiety are nearly coplanar. The dihedral angle between the two quinoxaline rings within each molecule is 9.22 (6)° and 2.45 (6)°, respectively. In the crystal packing, adjacent molecules are linked via pairs of intermolecular C–H ··· N interactions, forming R22(8) ring motifs and, together with other intermolecular C– ··· N interactions, assembled into chains propagating in [100]. Molecules are also stacked by π–π interactions between the pyrazine/pyrazine and benzene/benzene rings of adjacent sheets. The X-ray structure of DHQ has been reported with a CCDC number CCDC 977221.

### 2.2. Theoretical calculations

**Figure 4.**
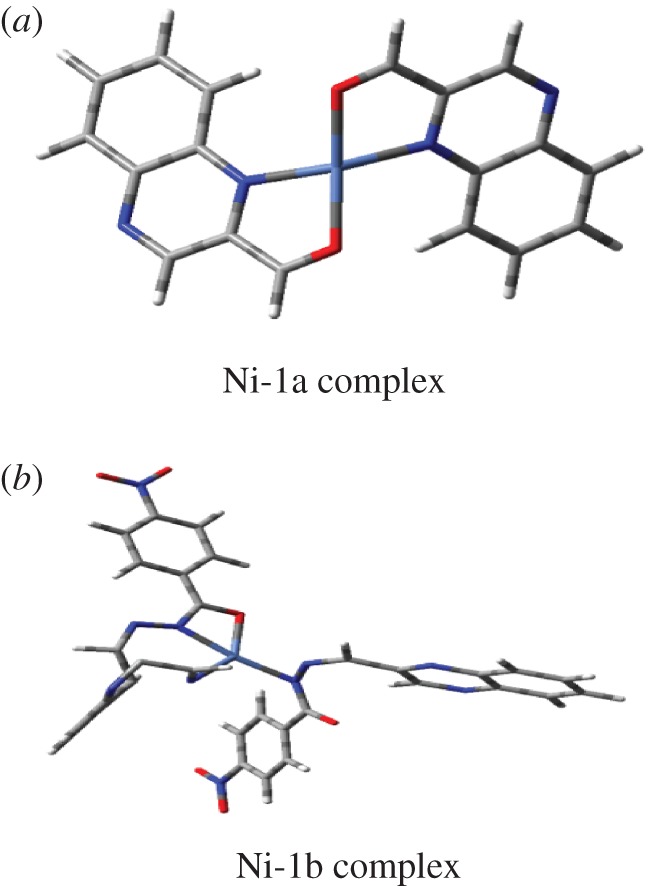
The structures of (*a*) Ni-QA (1a) and (*b*) Ni-HQNM (1b).

As in figure 4, DFT calculations were carried out using the Gaussian 03 (Revision B.04) [31–33] package. ‘Gauss View’ is used for visualization of molecular orbital (electronic supplementary material). The observation is that the compound QA (1a) does not bind with Ni^2+^. High dimerization stability of QA (1a) hydrogen bond formation and by the perfect stacking interactions with no steric crowding is the key reason for the formation of a highly stable stacked dimer. The calculated dimerization energy in 1a with that obtained from DFT calculations can be understood from the proposed less stable hindered chair forms of the nickel complex to the more open structure of the Ni–1a complex as shown above to be obtained by calculation.

**Figure 5.**
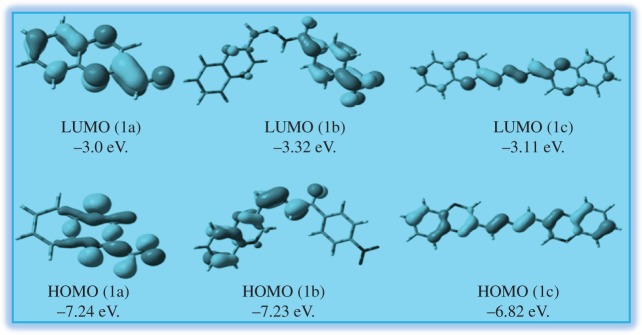
Frontier molecular orbital (HOMO and LUMO) of structures (1a) QA, (1b) HQNM and (1c) Quinoxaline DHQ with ISO value cut-off 0.04.

High dimerization stability of 1a by hydrogen bond formation and by the perfect stacking interactions with no steric crowding is the key reason for the formation of a highly stable stacked dimer. The calculated dimerization energy in the case of 1a is 3.5 and 8 kcal mol^−1^, respectively. The difference in the proposed complex structure of 1b with that obtained from DFT calculations can be understood from the proposed less stable hindered chair forms of the Ni^2+^ complex to the more open structure of the Ni-1b complex as shown above to be obtained by calculation (figure 5).

## 3. Conclusion

In conclusion, herein we report a new crystal structure for dimeric QA, HQNM and quinoxaline dihydrazone (DHQ). Among the three compounds, HQNM can selectively and successfully recognize nickel and DHQ recognizes Fe^3+^ cation selectively over other interfering cations in acetonitrile–HEPES buffer (9 : 1, v/v, pH = 7.4) solution, but QA cannot. The detection limits of Ni^2+^ and Fe^3+^ were found to be 1.47 µM and 1.60 × 10^−5^ M, respectively, from the absorption spectral change, which is sufficiently low and enables the detection of those cations in chemical and biological systems. The theoretical study of the three crystals along with the HOMO–LUMO calculation has also been shown.

## Supplementary Material

Supporting Information

## Data accessibility

This article does not contain any additional data.
