# Supplementary material for: Sensing study of quinoxaline analogues with theoretical calculation, single-crystal X-ray structure and real application in commercial fruit juices
Source: R Soc Open Sci. 2018 Jun 6;5(6):180149. doi: 10.1098/rsos.180149 (PMC6030317; doi:10.1098/rsos.180149)
Supplement: Supporting Information [file rsos180149supp1.docx]

Supporting Information

Sensing Study of Quinoxaline Analogues for Transition Metals with Theoretical calculation and Single Crystal X-Ray Structure

**CONTENTS**

| Sl no | Contents | Page no |
| --- | --- | --- |
| 1 | General Experimental | 2 |
| 2 | General procedure for drawing Job plot by UV–vis method | 5 |
| 3 | Determination of Detection Limit | 5 |
| 4 | Determination of Association Constant | 6 |
| 5 | ^1^H NMR of the compounds | 7 |
| 6 | Crystal Data | 9 |
| 7 | UV-vis titration spectra of HQNAP with Nickel chloride | 13 |
| 8 | UV-vis titration spectra of HQNM, DHQ and QA with different transition metals | 14 |
| 9 | Computational Details | 20 |
| 10 | References | 25 |

1. General Experimental:

All chemicals and solvents were purchased from Sigma-Aldrich chemicals Private Limited and were used without further purification. Melting points were determined on a hot-plate melting point apparatus in an open-mouth capillary and are uncorrected. 1H-NMR was recorded on Brucker 400 MHz instruments. For NMR spectra, DMSO-d^6^ was used as solvent using TMS as an internal standard. Chemical shifts are expressed in δ units and 1H–1H and 1H–C coupling constants in Hz. UV-vis titration experiments were performed on a JASCO UV-V630 spectrophotometer. IR spectra were recorded on a JASCO FT/IR-460 plus spectrometer, using KBr discs. 13C-NMR was recorded on a JEOL 500 MHz instrument.

**General method of UV-vis titration:**

For UV-vis titrations, stock solution of the sensor was prepared (c = 1 x 10-5 ML-1) in CH3CN. The solution of the guest cations using their salts in the order of 2 x 10-4 ML-1 was prepared in CH3CN solvent. Solutions of various concentrations containing sensor and increasing concentrations of cations were prepared separately. The spectra of these solutions were recorded by means of UV-vis methods.

**Synthesis of the ligands: 1.1 Synthesis of Quinoxaline aldehydes**

**1.1.1.Synthesis of 3,3 Dimethoxy 2-Oxo propanal (a)**

In a round bottom flask pyruvaldehyde dimethyl acetal (5.5 g, 46.5 mmol), Selenium dioxide (6.1 g, 55.8 mmol) and 1,4- Dioxane (20 ml) was taken and the reaction mixture was refluxed for 5h at 100^o^C. TLC checked, the starting was consumed then the reaction mixture was passed through Celite bed and the filtrate was collected. The reaction mixture was washed with chloroform to get the crude compound. The crude compound was directly take for the next step reaction.

**1.1.2 Synthesis of 2,2 Dimethoxy methyl Quinoxaline(b)**

The crude compound (6.1g, 46.55mmol) was dissolved in ethanol (50ml) and o-phenylene diamine (5g, 46.55mmol ) was added to it and the reaction mixture was refluxed for 12h at 80^o^C. The reaction mixture was cooled and the ethanol was evaporated by rotary evaporator and the crude material was purified by silica gel (100-200 mesh) column chromatography using (90/10, v/v) ethyl acetate-pet ether to afford a brown color solid (7.1g,74%).

**Scheme 1**: Synthesis of the Quinoxaline aldehyde

(C_11_H_12_N_2_O_2_): Color Brown solid. Mp 85 °C. ^1^H NMR (CDCl3, 400 MHz): δ (ppm): 9.10 (s, 1H), 8.17-8.12 (m, 2H), 7.80-7.77 (m, 2H), 5.56 (s, 1H), 3.50 (s, 6H).
**1.1 Synthesis of Quinoxaline aldehyde (1a)**

To the crude compound (4 g, 19.60 mmol) 2(N) HCl (19ml) was added at 0^o^C and the mixture was stirred for 48h at room temperature. The reaction mixture was quenched with saturated sodium carbonate solution and the mixture was extracted with ethyl acetate solution and finally purified by flash chromatography using 100-200 mesh silica-gel, pet ether as a eluting solvent. The product was eluted in ethyl acetate-pet ether (95/5, v/v) to afford a light yellow solid (2.6g, 86%)

(C_9_H_6_N_2_O); yellow solid, mp 109°C. ^1^H NMR (CDCl3, 400 MHz): δ (ppm): 10.19 (s, 1H), 9.36 (s, 1H), 8.31 (d, 1H, J=8.3Hz), 8.24 (d, 1H, J=8.3Hz ), 7.89-7.82 (m, 2H).

**1.2 Synthesis of HQNM(1b) :**

Scheme 2: Synthesis of the Receptor HQNM

To a stirred solution of quinoxaline aldehydes (17mg, 0.10mmol) in 1 ml of methanol solution, the amine X [(19mg, 0.10 mmol) dissolved in 0.5 ml of methanol] was added. A precipitate was appeared instantaneously. The reaction mixture was stirred for another one hour.TLC shows the completion of the reaction. The product was filtered and washed with little amount of ethanol two times.

(C_16_H_11_N_5_O_3_): Yield 67%. Color Yellow. mp >280 °C.

^1^H NMR (DMSO-d6 ,400 MHz): δ (ppm): 12.6134(s, 1H), 9.4850 (s, 1H), 8.6680 (s, 1H,), 8.4347 (d, 1H, J=8.24), 8.4141 (d, 2H, J=8.24) ,8.1980 (t, 2H J= 8.40), 8.1472(s, 3H), 7.9037 (d,1H, J=4.92).
**MS** : 322(M+1).

**^13^C NMR (DMSO-d6, 500 MHz):** δ (ppm):157.3042, 152.8120, 149.6349, 145.0083, 142.6374, 141.7543, 136.2831, 131.9061, 131.4645, 130.1879, 129.8711, 129.5640, 120.3300, 120.0317, 117.2105.

**Synthesis of HQNM-Ni Complex**

To a hot 1.0 ml methanolic solution containing 10mg (0.032 mmol) of the ligand, 1.0 mL of a methanolic solution containing 10.39 mg (0.064 mmol) of NiCl_2_.6H_2_O was added. A yellow precipitate appears immediately. After stirring for 1.0 h the yellow complexes were filtered, collected and then washed for several times with cold methanol. The complexes were dried in a desiccator over anhydrous CaCl_2_ under vacuum. The dried ligands and complexes were subjected to spectroscopic analyses. The complexes are air-stable, non-hygroscopic, soluble in ethanol, methanol, DMSO, DMF.

NiL_2_ (C30H20N6O2Ni): Yield 80%. Colour: yellow. mp >280 °C.

MS (FD):700 (2 HNQM+Ni).

**1.3 Synthesis of dihydrazone (DHQ) of quinoxaline aldehyde(1c)**

Scheme 3: Synthesis of the Receptor DHQ

To a stirred solution of quinoxaline aldehydes (50mg, 0.31mmol) in 1 ml of methanol solution, the hydrazine hydrate [(20.2 mg, 0.63 mmol) dissolved in 0.5 ml of methanol] was added. A precipitate appeared instantaneously. The reaction mixture was stirred for another one hour. TLC showed the two different spots along with some left over starting material. The product was filtered and washed with little amount of ethanol two times. Then the two compounds was separated by column chromatography using silica-gel (100-200 mesh) in 5% EtoAC-Hexane solution as eluting solvent.

**Synthesis of DHQ-Fe Complex**

To a hot 1.0 ml methanolic solution containing 20mg (0.06 mmol) of the ligand, 1.0 mL of a methanolic solution containing 7.3 mg (0.03 mmol) of FeCl_3_.6H_2_O was added. A yellow precipitate appears immediately. After stirring for 1.0 h the yellow complexes were filtered, collected and then washed for several times with cold methanol. The complexes were dried in a desiccator over anhydrous CaCl_2_ under vacuum. The dried ligands and complexes were subjected to spectroscopic analyses. The complexes are air-stable, non-hygroscopic, soluble in ethanol, methanol, DMSO, DMF.

FeL_2_ (C_36_H_24_N_12_Fe): Yield 75%. Colour: yellow. mp >280 °C.

MS (FD):679 (2 DHQ+Fe).

**2. General procedure for drawing Job plot by UV–vis method**

Stock solution of same concentration of sensor and Fe3+ were prepared in the order of ≈ 1.0 x 10-5 ML-1 CH3CN . The absorbance in each case with different host–guest ratio but equal in volume was recorded. Job plots were drawn by plotting ∆I.Xhost vs Xhost (∆I = change of intensity of the absorbance spectrum during titration and Xhost is the mole fraction of the host in each case, respectively).

**Figure 1:** Job’s plot diagram of receptor **DHQ** for Fe^3+^ cation (where Xh is the mole fraction of host and ΔI indicates the change of the absorbance).

**3. Determination of Detection Limit:**

The detection limit DL of DHQ for Ni^+2^ was determined from the following equation1:

DL = K* Sb1/S. Where K = 2 or 3 (we take 3 in this case); Sb1 is the standard deviation of the blank solution; S is the slope of the calibration curve. From the graph we get slope = 19133 , and Sb1 value is. 0.005534. Thus using the formula we get the Detection Limit = 1.60x10^-5^ M i.e. **DHQ** can detect Fe^3+^ in this minimum concentration.

In similar way we have calculated the Detection Limit 1.47 µM for HQNM in our previous communication.^1a^

**4. Determination of Association constant:**

Binding constant was calculated for DHQ according to the Benesi-Hildebrand equation. Ka was calculated following the equation stated below.

1/(A-Ao) = 1/{K(Amax–Ao) [Ni2+]n} + 1/[Amax-Ao]

Here Ao is the absorbance of receptor in the absence of guest, A is the absorbance recorded in the presence of added guest, Amax is absorbance in presence of added [Fe^3+]^max and K is the association constant (M-1). The association constant (K) could be determined from the slope of the straight line of the plot of 1/(A-Ao) against 1/[Fe^3+]^n. The association constant (Ka) as determined by UV-vis titration method for sensor with Fe3+ is found to be 2.5×10^5^ M^-1^.

**Figure S1:** Benesi–Hildebrand plot from UV-vis titration data of receptor **DHQ** (1x10^-5^M) with Fe^3+^.

In similar method, the association constant^1a^ (*K_a_*) was calculated by UV-vis titration method for sensor **HQNM** with Ni^2+^ is found to be 2.5×10^5^ M^-1^.

**^1^H NMR of the compounds**

**^1^H NMR of quinoxaline aldehyde**:


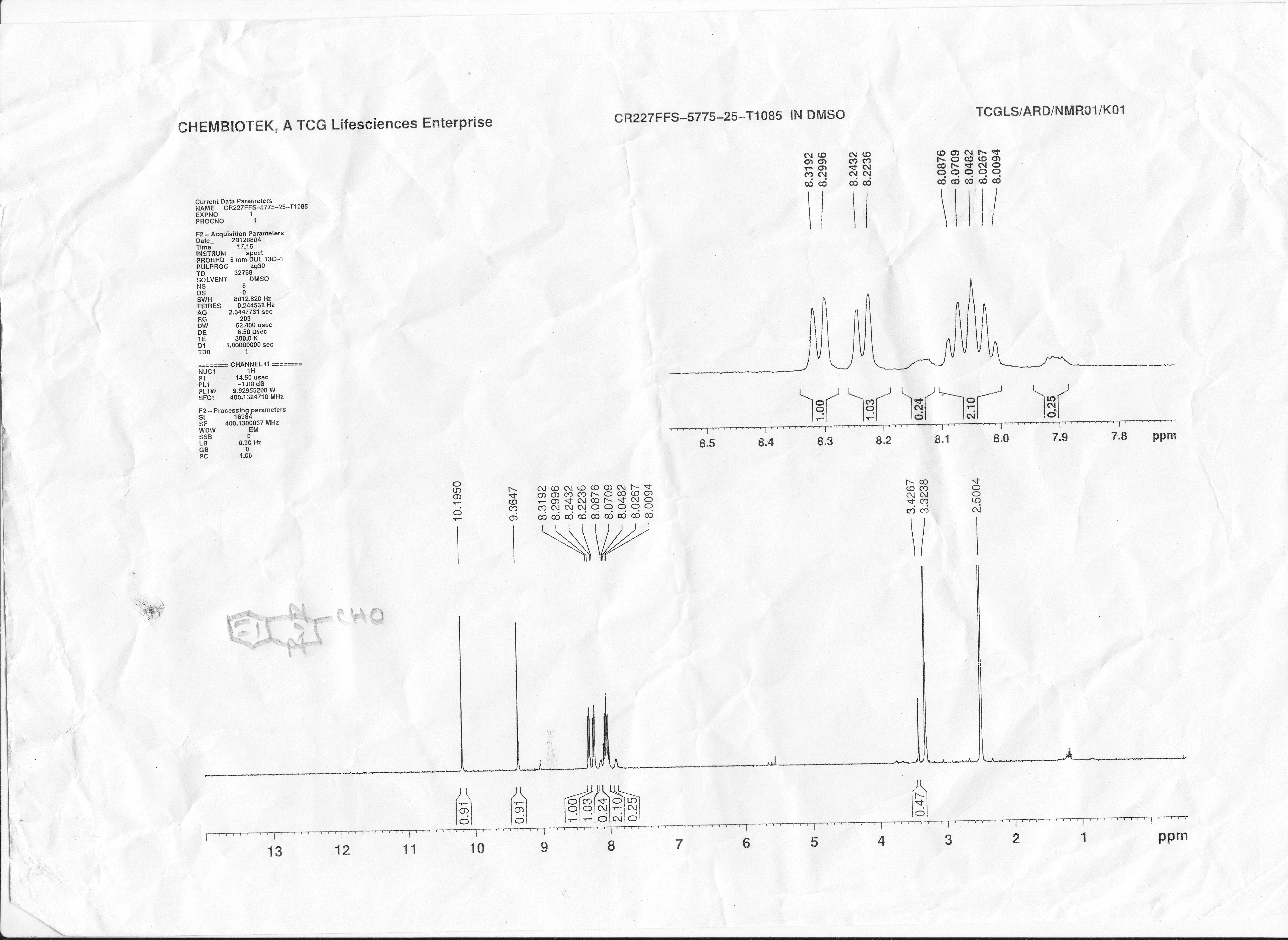


**^1^H NMR of HQNM**:

**^13^C NMR of HQNM:**

**
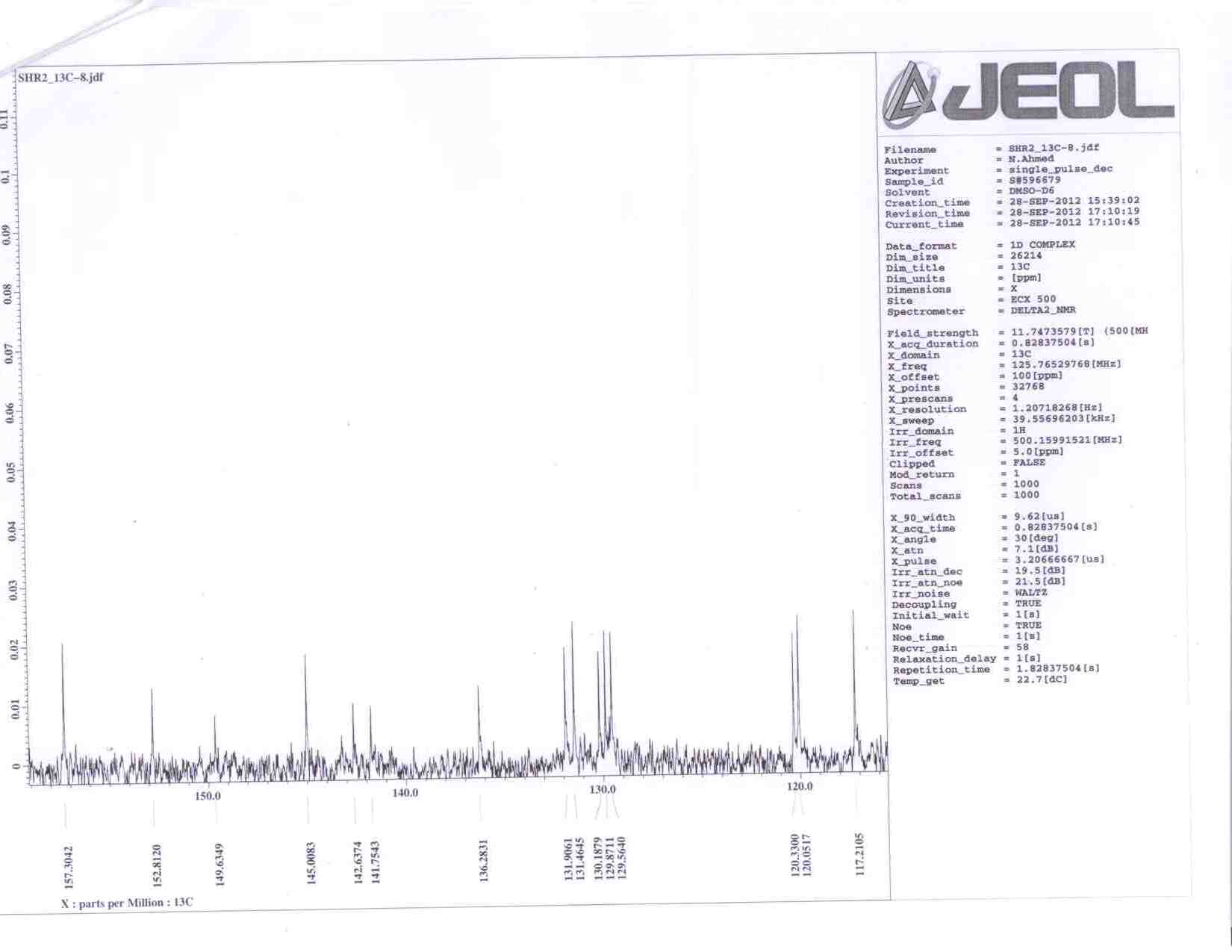
**

**^1^H NMR of DHQ**

**
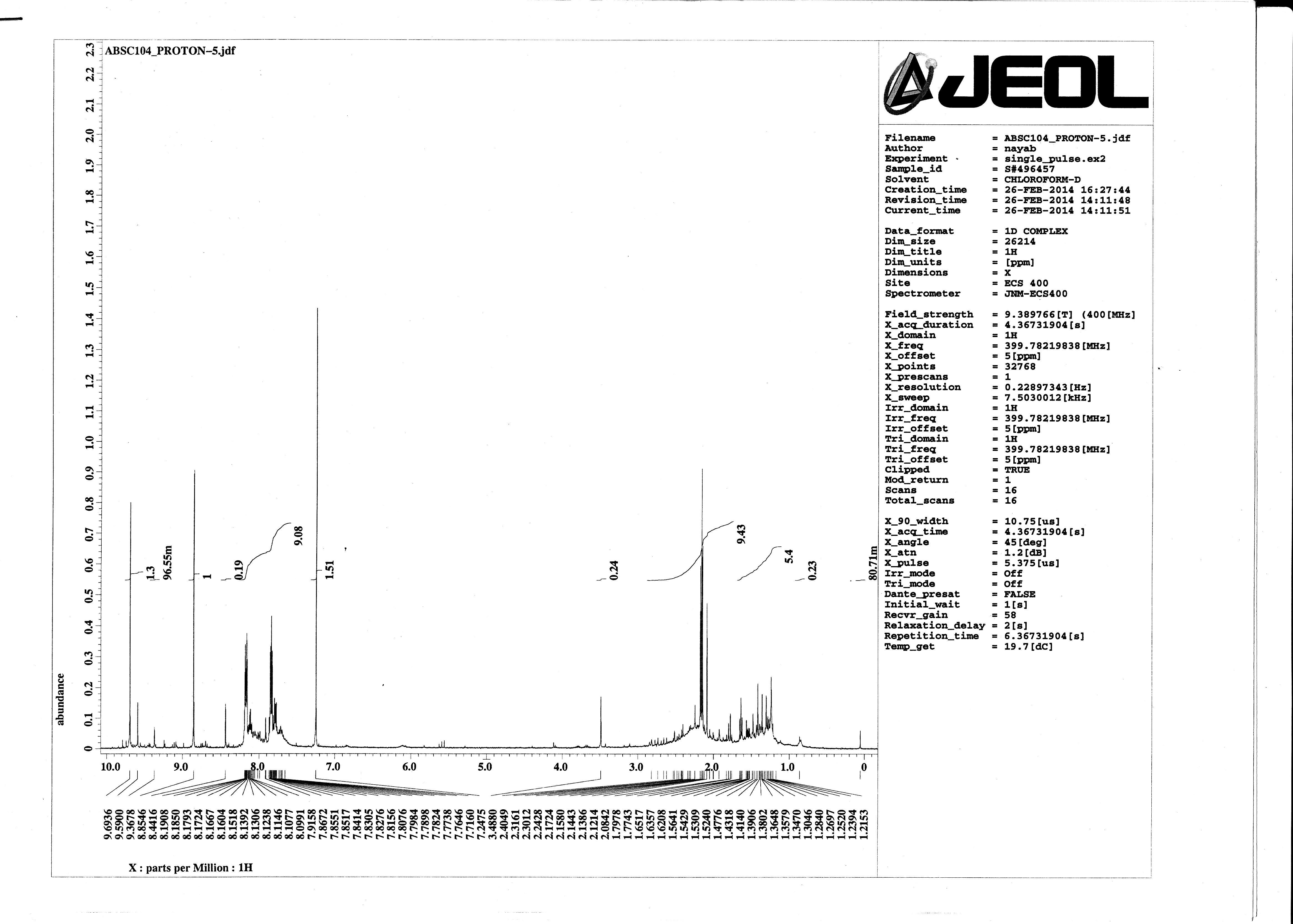
**

**^13^ C NMR of DHQ**

**
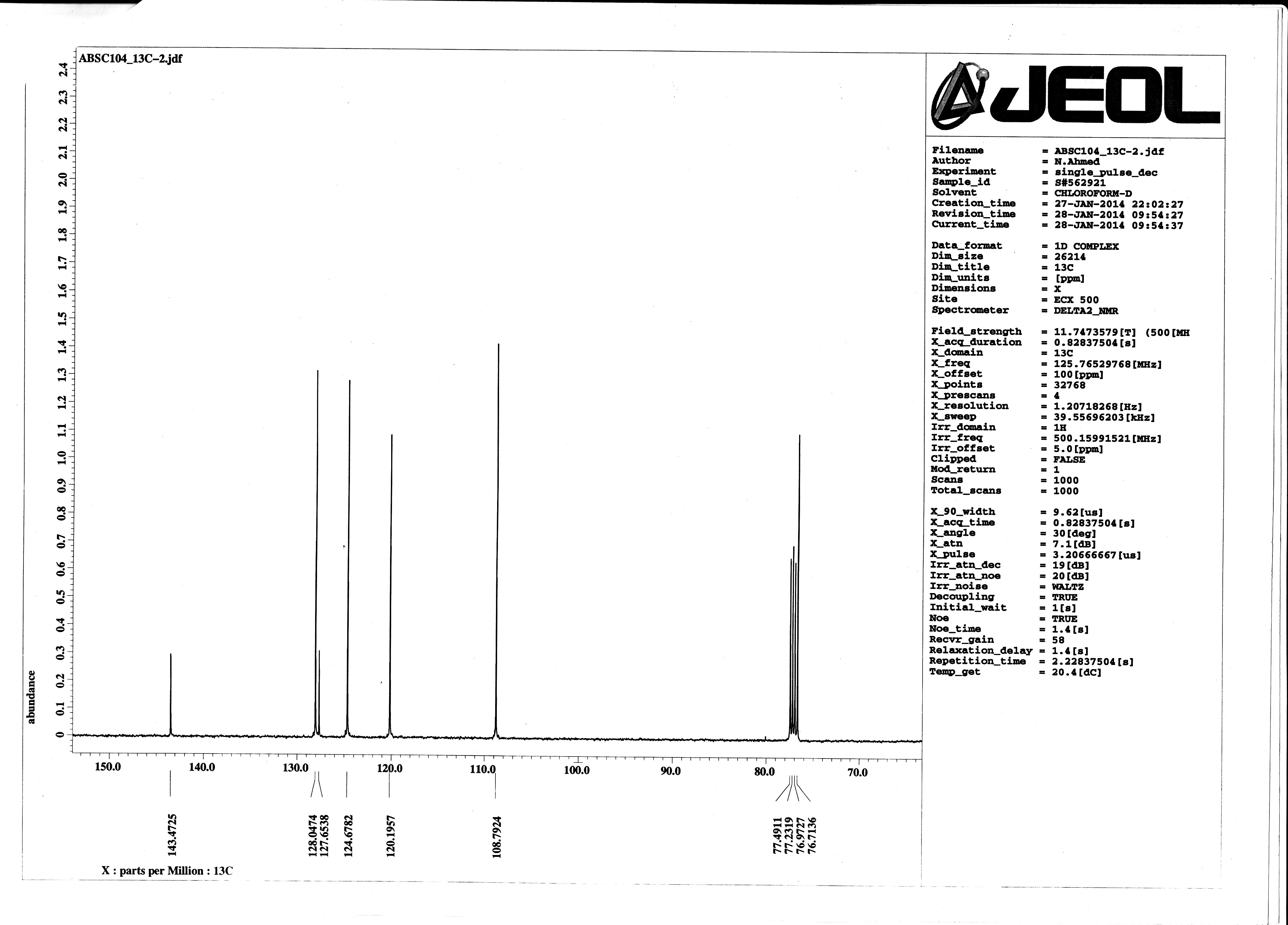
**

**6. Crystal Data:**

X-ray diffraction data of single crystals of HQNM(with dimensions of 0.64 × 0.17 × 0.09 mm), DHQ (with dimensions of 0.698×0.144×0.064 mm) and quinoxaline aldehyde (with dimensions of 0.607×0.488×0.317 mm) were collected on Bruker APEX II or APEX II Duo CCD area-detector diffractometer operating at 50kV and 30mA using Mo Kα radiation (λ = 0.71073 Å). Diffraction data were collected with the Oxford Cryosystem Cobra low temperature attachment at 100.0 (1) K^1^ . Data collection and reduction were performed using the APEX2 and SAINT software^2^. The SADABS software was used for absorption correction ^2^. HQNM, DHQ and quinoxaline aldehyde were solved by direct method and refinement was carried out by the full-matrix least-squares technique on F^2^ using SHELXTL package ^3^. All non-hydrogen atoms were refined anisotropically whereas hydrogen atoms were refined isotropically. N-bound H atoms were located in the difference Fourier map and refined freely [N—H = 0.90(2) and 0.93(2) Å]. The remaining H atoms were placed in calculated positions with O—H = 0.85 Å and C—H = 0.93 or 0.95 Å after checking their positions in the Fourier difference map. The U_iso_ values were constrained to be 1.2 U_eq_ of the carrier atom. The crystallographic data and hydrogen bonds geometry are presented in Table 1 and 2, respectively. Crystallographic data for HQNM, DHQ and quinoxaline aldehyde have been deposited with the Cambridge Crystallographic Data Center No. CCDC 1023223, CCDC 977221 and CCDC 978283, respectively. Copy of the data can be obtained free of charge on application to the CCDC, 12 Union Road, Cambridge CB2 IEZ, UK. Fax: +44-(0)1223-336033 or E-Mail: [deposit@ccdc.cam.ac.uk](mailto:deposit@ccdc.cam.ac.uk).


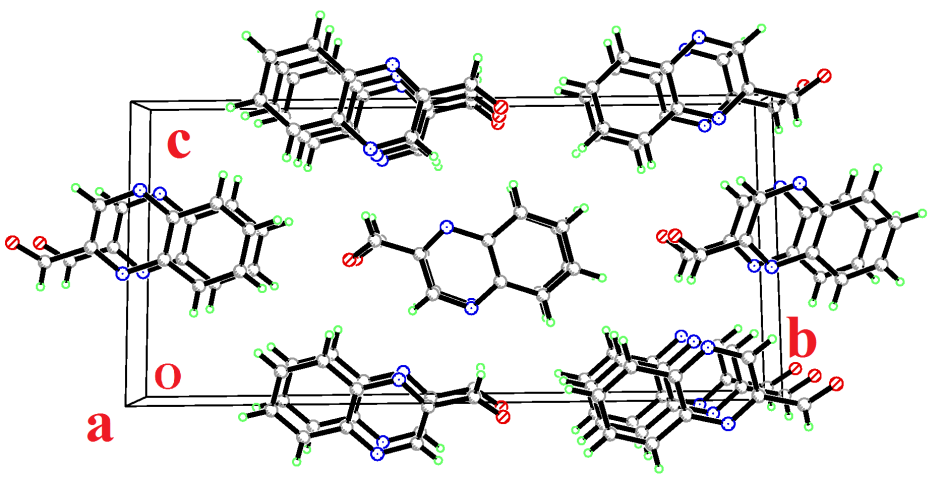


**Figure 5. The crystal packing of quinoxaline aldehyde (QA) viewed along the *a* axis.**

| 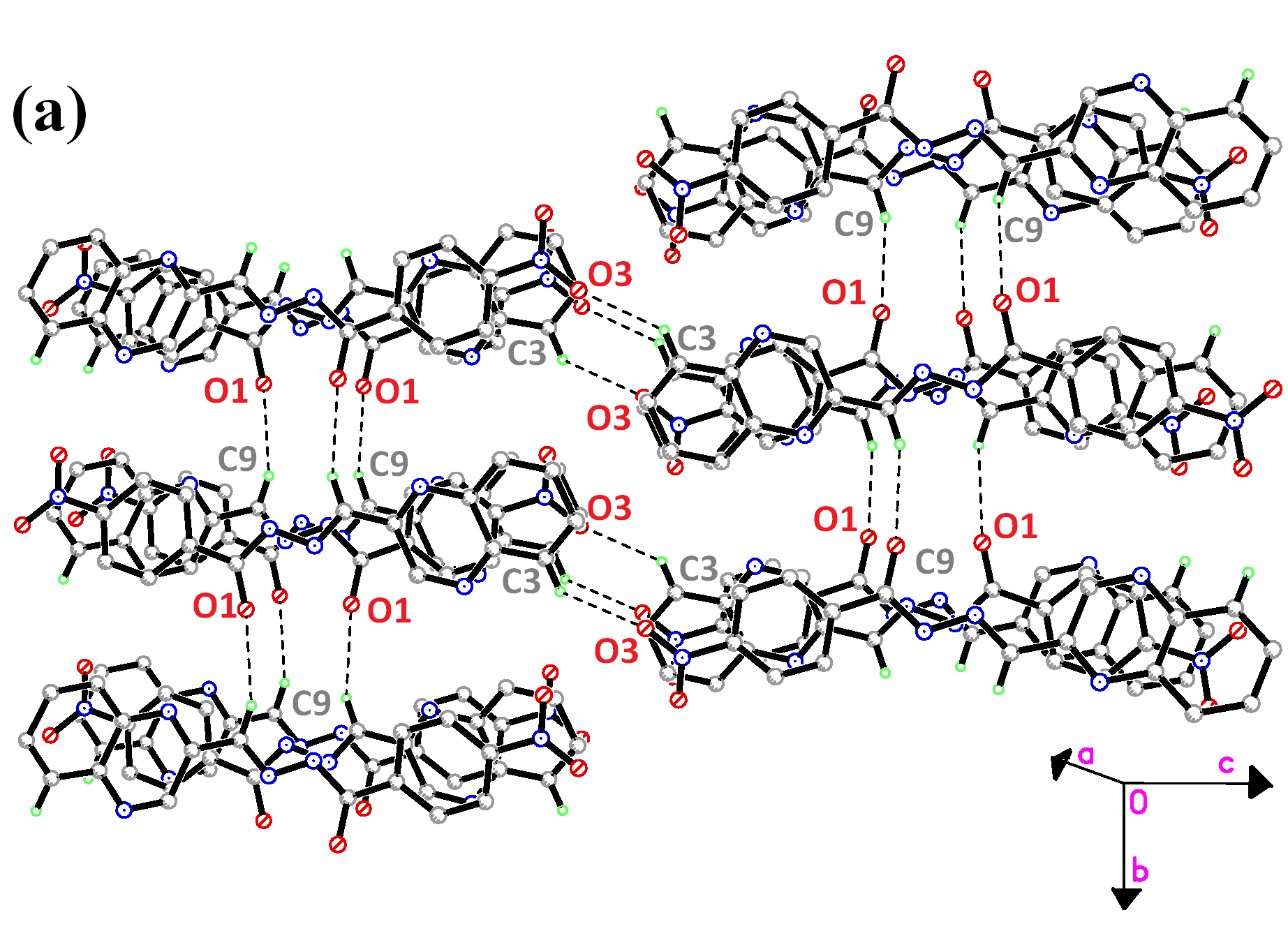 | 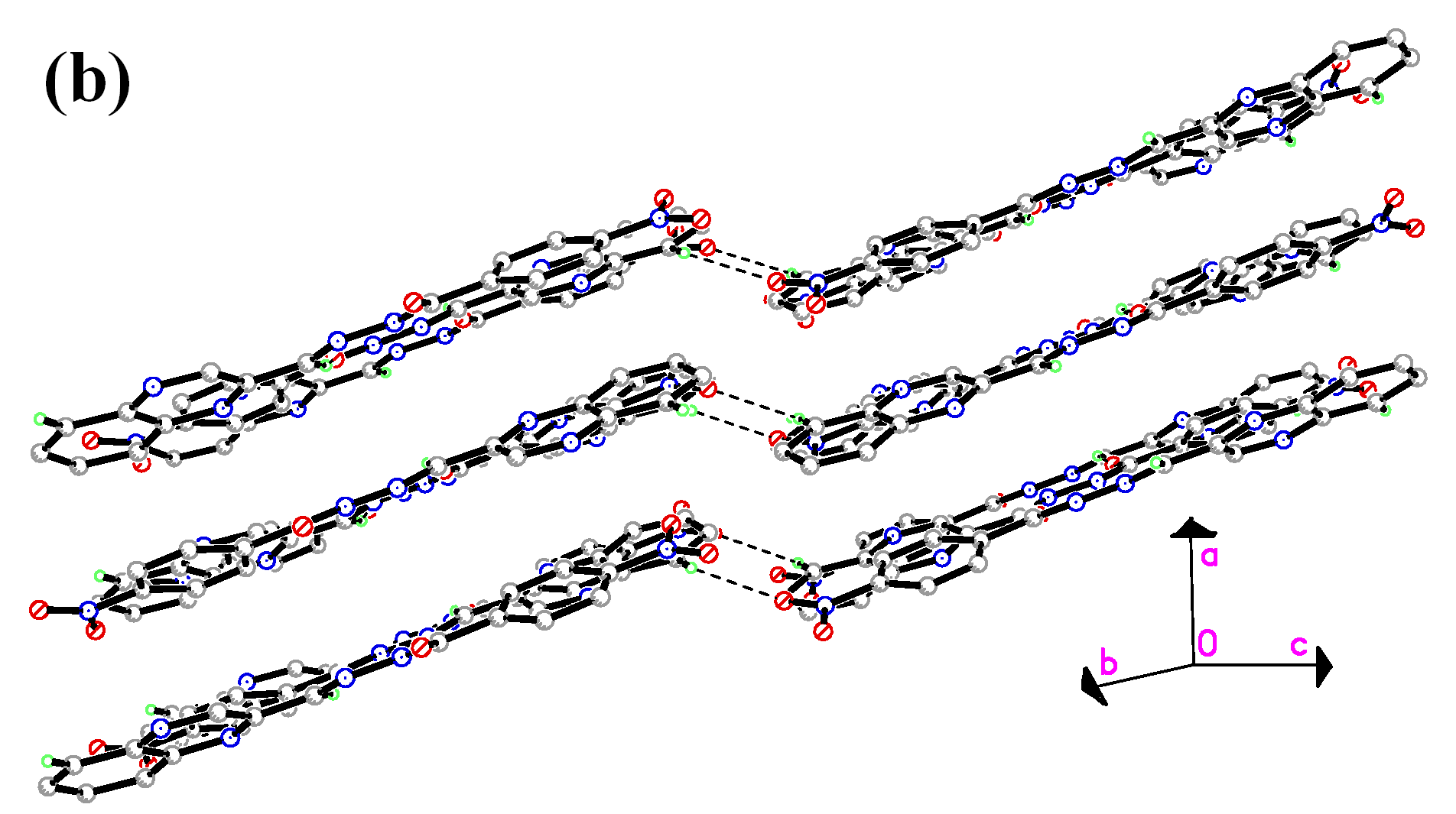 |
| --- | --- |
| 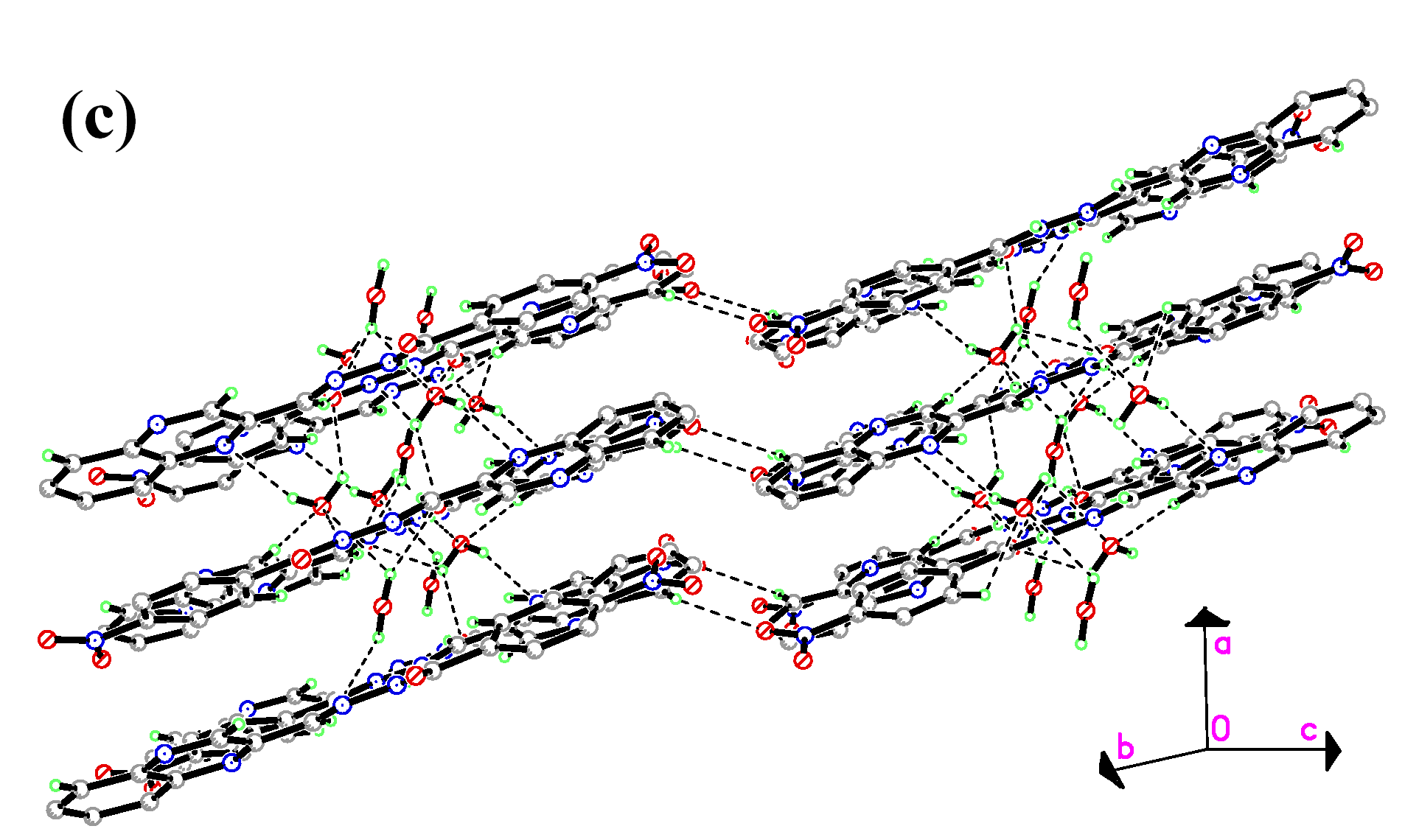 | |
| **Figure 6. Part of the crystal packing of HQNM(excluding water molecules) viewed along (a) [100] and (b) [010]. (c) Crystal packing of HQNM showing three-dimensional network. H atoms not involved in intermolecular interactions (dashed lines) have been omitted for clarity.** | |

| 1. 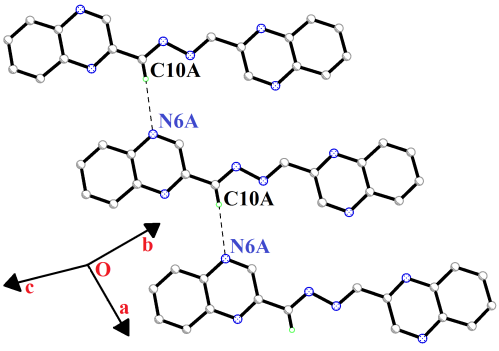 | 1. 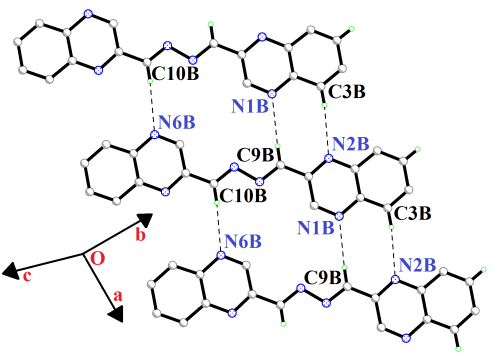 |
| --- | --- |
| 1. 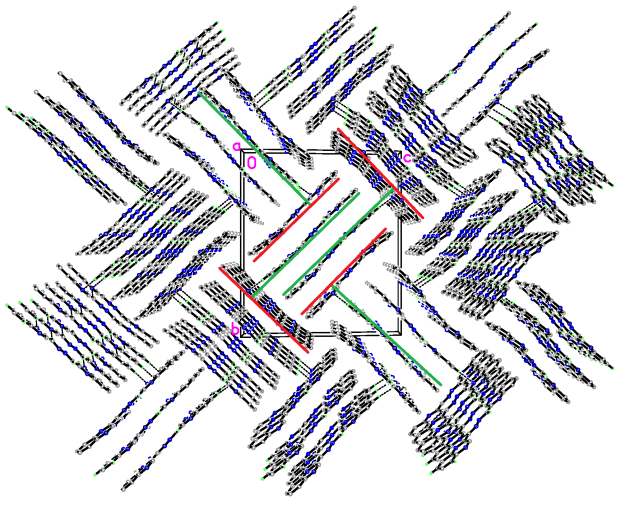 | |
| **Figure 7. Part of the crystal packing of (a) molecules *A* (b) molecules *B*, viewed along the *bc* axis. (c) The crystal packing of DHQ viewed along the *a* axis. Molecules *A* and *B* are indicated by red and green lines, respectively. H atoms not involved in intermolecular interactions (dashed lines) have been omitted for clarity.** | |

**Table 1**: X-ray crystallographic data

| **Compounds** | **Quinoxaline aldehyde**  (CCDC 978283) | **HQNM**  (CCDC 1023223) | **DHQ**  (CCDC 977221) |
| --- | --- | --- | --- |
| Formula | C_9_H_6_N_2_O | 2(C_16_H_11_N_5_O_3_)·3(H_2_O) | C_18_H_12_N_6_ |
| Formula Weight | 158.16 | 696.64 | 312.34 |
| Crystal System | Monoclinic | Monoclinic | Monoclinic |
| Space Group | *P*[2_1_](file:///C:\Users\Shampa\Desktop\Paper\Crystal\Full%20Paper\Downloads\spgfun10_0m%20_symmetry_space_group_name_H-M) | *C*2/*c* | *P*[2_1_/c](file:///C:\Users\Shampa\Desktop\Paper\Crystal\Full%20Paper\Downloads\spgfun10_0m%20_symmetry_space_group_name_H-M) |
| T (K) | 100 | 100 | 100 |
| Z | 4 | 4 | 8 |
| a (Å) | 3.7476(1) | 7.5271 (14) | 6.6927 (10) |
| b (Å) | 20.2550(6) | 12.106 (2) | 22.521 (3) |
| c (Å) | 9.6237(3) | 33.583 (7) | 19.677 (3) |
| β (deg) | 98.845(1) | 91.284 (12) | 101.350 (5) |
| V (Å^3^) | 721.83(4) | 3059.4 (10) | 2907.8 (7) |
| d_calcd_ (g/cm^3^) | 1.455 | 1.512 | 1.427 |
| µ (mm^-1^) | 0.10 | 0.11 | 0.09 |
| Reflections with *I* > 2*σ*(*I*) | 1635 | 1366 | 3172 |
| Independent reflections | 1707 | 2428 | 5074 |
| θ range (deg) | 2.37–27.56 | 2.4–24.3 | 1.40–25.0 |
| *hkl* range | *h* = −4→3  *k* = −26→24  *l* = −12→12 | *h* = −8→8  *k* = −13→11  *l* = −36→38 | *h* = −7→7  *k* = −21→26  *l* = −23→23 |
| GOF (F^2^) | 1.05 | 1.08 | 1.03 |
| R_1_, wR_2_ (%) | 0.035, 0.094 | 0.131, 0.401 | 0.047, 0.152 |
| Completeness (%) | 99.7 | 0.977 | 99.7 |
| *T*_min_, *T*_max_ | 0.915, 0.969 | 0.9303, 0.9900 | 0.939, 0.994 |

**Table 2 :** Hydrogen-bond geometry (Å, º)

| ***D*—H···*A*** | ***D*—H** | **H···*A*** | ***D*···*A*** | ***D*—H···*A*** |
| --- | --- | --- | --- | --- |
| **Compound HQNM** |  |  |  |  |
| O1*W*—H1*W*1···O1^i^ | 0.85 | 2.17 | 2.981 (9) | 160 |
| O2*W*—H1*W*2···O1^ii^ | 0.85 | 2.34 | 2.959 (10) | 129 |
| O2*W*—H2*W*2···N2^iii^ | 0.85 | 2.18 | 2.976 (9) | 157 |
| N4—H4*B*···O2*W*^iv^ | 0.88 | 2.01 | 2.836 (9) | 156 |
| C1—H1*A*···O2*W* | 0.95 | 2.38 | 3.328 (11) | 176 |
| C3—H3*A*···O3^v^ | 0.95 | 2.53 | 3.302 (9) | 139 |
| C9—H9*A*···O1^iv^ | 0.95 | 2.56 | 3.461 (9) | 158 |
| C12—H12*A*···O2*W*^iv^ | 0.95 | 2.53 | 3.157 (10) | 124 |
| **Compound DHQ** |  |  |  |  |
| C3*B*—H3*BA*···N2*B*^vi^ | 0.93 | 2.58 | 3.492 (3) | 167 |
| C5*B*—H5*BA*···N6*A*^vii^ | 0.93 | 2.50 | 3.375 (3) | 156 |
| C10*A*—H10*A*···N6*A*^vi^ | 0.93 | 2.57 | 3.485 (3) | 167 |
| C10*B*—H10*B*···N6*B*^vi^ | 0.93 | 2.59 | 3.509 (3) | 170 |
| C9*B*—H9*BA*···N1*B*^viii^ | 0.93 | 2.52 | 3.450 (3) | 175 |

Symmetry codes: (i) −x+1, y, −z+1/2; (ii) −x, y, −z+1/2; (iii) x−1/2, y+1/2, z; (iv) −x+1/2, y−1/2, −z+1/2; (v) x+1/2, −y+1/2, z+1/2; (vi) *x*−1, *y*, *z*; (vii) −*x*+2, *y*−1/2, −*z*+3/2; (viii) *x*+1, *y*, *z*.

7. **UV-vis titration spectra of HQNAP with Nickel chloride**

**Figure 8**: UV–vis absorption spectra of HQNAP (1×10-5 M) in CH3CN-HEPES buffer (9:1, v/v, pH=7.4) upon titration with nickel chloride (NiCl_2_.6H_2_O, 0.8 equiv). The arrows show changes due to the increasing concentration of Ni^2+.^

**8.1 UV-vis titration spectra of HQNM (*c* = 1.0 x 10^–5^ M) with another cations except Ni^2+^ i.e. (*c* = 2.0 x 10^–4^ M) in CH_3_CN-HEPES buffer (9:1, v/v, pH=7.4) .**

|  |  |
| --- | --- |
|  |  |
|  |  |
|  |  |
|  |  |

**8.2 UV-vis titration spectra of DHQ (*c* = 1.0 x 10^–5^ M) with another cations except Fe^3+^ i.e. (*c* = 2.0 x 10^–4^ M) in CH_3_CN-HEPES buffer (9:1, v/v, pH=7.4) .**

|  |  |
| --- | --- |
|  |  |
|  |  |
|  |  |
|  |  |
|  |  |

**8.3 UV-vis titration spectra of QA (*c* = 1.0 x 10^–5^ M) with all probable cations (*c* = 2.0 x 10^–4^ M) in CH_3_CN-HEPES buffer (9:1, v/v, pH=7.4) .**

|  |  |
| --- | --- |
|  |  |
|  |  |
|  |  |
|  |  |

**8.4 Blank titration (Without Receptor DHQ) with Pomegranate and Grapes Juices**

|  |  |
| --- | --- |
| **Figure 9a:** UV–vis absorption spectra of CH_3_CN-HEPES buffer upon titration with Commercial Grapes juices. The arrows show changes due to the increasing concentration of Fe^3+^. | **Figure 9b:** UV–vis absorption spectra of CH_3_CN-HEPES buffer upon titration with Commercial Pomegranate juices. The arrows show changes due to the increasing concentration of Fe^3+^. |
|  |  |

**9. Computational details:**

DFT calculations were carried out using the Gaussian 03 (Revision B.04)* package. “Gauss View” is used for visualization of Molecular orbital. Becke’s three parameter hybrid exchange functional,^4^ the nonlocal correlation provided by the Lee, Yang, and Parr expression, and the Vosko, Wilk, and Nusair 1980 correlation functional (III) local (B3LYP) 6-311+g(d,p) basis set^5^ were used for H, C, N and O atoms without any further modification for optimizing all ligand structures with no symmetry constrain and the initial geometry were taken from crystal structure. The LANL2DZ^6^ basis set and LANL2DZ pseudopotentials of Hay and Wadt^7^ were used for the Ni atom while optimizing the Ni – ligand complexes.

Optimized Coordinates:

Ligand 1-a

O 4.04722700 -0.02905600 -0.00016100

N 0.68937600 -1.09009100 0.00004100

N 0.18779700 1.70139200 0.00005700

C -0.58388500 -0.61328800 0.00001700

C -1.67511700 -1.52244800 -0.00003400

H -1.45584700 -2.58531100 -0.00002000

C -2.96616600 -1.04188800 -0.00006200

H -3.80291000 -1.73375400 -0.00015000

C -3.21761000 0.35615200 -0.00002800

H -4.24366500 0.71165800 -0.00002200

C -2.17760600 1.26197000 0.00001900

H -2.34805100 2.33362200 0.00000900

C -0.83814200 0.79868100 0.00007400

C 1.40769200 1.21082600 -0.00008400

H 2.24330500 1.90548700 0.00005200

C 1.66162900 -0.19404700 0.00006400

C 3.05265800 -0.72971000 0.00007900

H 3.10843300 -1.83585000 0.00047100

Ligand 1-b

C 3.18081300 -1.16432100 -0.53330700

C 4.38931300 -0.92898000 0.11092100

C 4.70234900 0.30633300 0.66995700

C 3.77004400 1.33109400 0.58277900

C 2.53398400 1.11869100 -0.03938900

C 2.25211800 -0.13153900 -0.60439800

H 2.98526300 -2.13335700 -0.97091300

H 5.65784100 0.44879300 1.15543100

H 3.98565900 2.31051400 0.99024100

H 1.32361000 -0.30777500 -1.13420700

N 5.37916800 -2.02876800 0.19731800

O 5.07879100 -3.10206800 -0.30880200

O 6.43448000 -1.79637400 0.77094800

C 1.59059000 2.29310400 -0.09972000

O 1.98636200 3.43541100 -0.08297600

N 0.24539200 1.94222700 -0.17655100

H -0.01029000 0.97831800 0.00951700

N -0.70158600 2.91760000 -0.13596500

C -1.94435000 2.60236700 -0.14062400

H -2.62555000 3.43993100 -0.02015900

C -2.59314500 1.27255600 -0.24585900

C -2.13072200 0.25231600 -1.13342900

C -4.31636200 -0.09717100 0.38631500

H -1.28054100 0.43904900 -1.78388800

C -5.48278800 -0.33665800 1.15653800

C -3.81964000 -1.12739300 -0.47285100

C -6.11434200 -1.55377200 1.07800900

H -5.84327400 0.45813700 1.79830000

C -4.49344200 -2.37099800 -0.53354500

C -5.61711900 -2.57619000 0.23044500

H -7.00365100 -1.74038900 1.66887800

H -4.09931300 -3.13510200 -1.19266100

H -6.13499800 -3.52729200 0.18612800

N -3.68755200 1.10176200 0.47455000

N -2.72051300 -0.91328100 -1.24664900Ligand 1-c

N 4.57215000 -1.74911400 -0.14929300

N 3.90255200 1.00630600 -0.04269100

N 0.62887600 -0.33202600 -0.03734700

N -0.62636800 0.30688900 -0.07451200

N -3.91388200 -1.01932600 0.07516700

N -4.54599700 1.74033100 -0.13426000

C 3.32885700 -1.32847300 -0.20538400

H 2.57073300 -2.03413600 -0.29668400

C 5.54477000 -0.78533600 -0.03683000

C 6.90203100 -1.17636900 0.03161300

H 7.14885600 -2.18425700 -0.02246300

C 7.88306800 -0.21949400 0.16912600

H 8.88127800 -0.50517300 0.21986000

C 7.54240600 1.15844800 0.24196800

H 8.29459600 1.86696800 0.35456400

C 6.22828200 1.56411700 0.16680600

H 5.98263900 2.57266600 0.21317200

C 5.20220000 0.59619200 0.02630400

C 2.98117700 0.06237600 -0.14611900

C 1.58962300 0.51409200 -0.18495700

H 1.38634300 1.52503600 -0.33327300

C -1.58859500 -0.54732800 0.00454100

H -1.38918300 -1.56646500 0.08770200

C -2.97814600 -0.08654000 -0.02245000

C -5.20951800 -0.59332800 0.05841200

C -6.25885100 -1.54752500 0.11956300

H -6.02908200 -2.56085800 0.13731600

C -7.56824000 -1.12738000 0.15434500

H -8.33401500 -1.82761200 0.21068200

C -7.88428200 0.26027700 0.11457300

H -8.87749200 0.56236500 0.16251300

C -6.88717700 1.20406600 0.01352900

H -7.11795400 2.21578800 -0.03997000

C -5.53492200 0.79251400 -0.01876800

C -3.30901300 1.30413500 -0.13966200

H -2.54003800 1.99760300 -0.23251400

(Ni-1a complex)

Ni -0.37077100 0.40292800 2.47580900

C -2.50448300 1.44675300 0.41359900

C -1.85243800 -0.78400400 0.38299300

C -2.45830500 2.77250100 0.91623500

C -3.42140200 1.12863100 -0.68280300

C -2.75425300 -1.05929800 -0.66558300

C -3.27067500 3.73295300 0.36219600

H -1.79985300 3.00941000 1.74340300

C -4.23621800 2.15479200 -1.22481700

H -2.84156100 -2.06542000 -1.07127500

C -4.16028400 3.42836700 -0.71286800

H -3.24361900 4.74703700 0.74885900

H -4.90726500 1.89254200 -2.03578300

H -4.78421900 4.21728400 -1.12098100

C 2.70864400 0.86467400 2.03236100

C 1.52332800 2.24109700 3.48247400

C 2.81765200 -0.20732200 1.10983300

C 3.90740200 1.62565600 2.39006300

C 2.69852000 2.94694500 3.81317800

C 4.04392600 -0.50838700 0.56674900

H 1.93910800 -0.78945800 0.85865800

C 5.14960600 1.27982200 1.80011400

H 2.67001200 3.76537600 4.53001500

C 5.21409300 0.23635300 0.90748000

H 4.13277200 -1.33263300 -0.13419800

H 6.02241600 1.85854200 2.08303300

H 6.16270600 -0.03539200 0.45524200

N -1.72920900 0.45630800 0.91244600

N -3.51642600 -0.11611200 -1.19057300

N 1.53108600 1.21377200 2.60018800

N 3.86467900 2.64594000 3.26947600

C -0.99293900 -1.79850800 0.97338500

O -0.20040400 -1.49997500 1.88908900

H -1.02917600 -2.83348000 0.61541400

C 0.22388000 2.55737100 4.05506000

O -0.78904500 1.91253300 3.71754400

H 0.12130800 3.36934600 4.78361300

(Ni-1b complex)

C 5.05035900 -1.34658000 5.41589600

C 5.74919300 -0.25053000 5.82956300

C 5.80991100 0.88814100 4.96277900

C 5.22139200 0.83463000 3.54696100

C 4.44619200 -0.38025900 3.22670500

C 4.37528000 -1.39413400 4.12569700

H 4.97288400 -2.21476800 6.06179500

H 6.23343300 -0.19865200 6.79758200

H 3.93824300 -0.44885000 2.26973400

H 3.80566600 -2.28635800 3.88701700

C 6.86266300 2.30575600 2.90889200

C 6.01516500 2.85297300 4.02270800

H 7.70467200 2.77846600 2.42274600

N 6.28747400 2.09114000 5.21778500

N 6.39488800 1.14346900 2.54799500

C 4.54811000 2.29702500 3.57035900

H 3.86932400 2.41721600 4.41523100

N 3.86049800 2.76139500 2.39913600

C 3.72885600 2.50538400 0.01629100

O 4.32085300 1.81394300 -0.91240200

N 4.29798200 2.25227500 1.24064500

C 2.61594300 3.41337200 -0.30739100

C 1.94475400 4.19403400 0.65748000

C 2.22292300 3.48866600 -1.66192600

C 0.89950600 5.03296700 0.27210500

H 2.24680700 4.13308800 1.69353500

C 1.18119100 4.32374600 -2.05113800

H 2.74929600 2.88487300 -2.39030400

C 0.53342100 5.08555400 -1.07392500

H 0.36995100 5.64111300 0.99318700

H 0.86533300 4.39615500 -3.08312000

N -0.56688400 5.96941600 -1.47712100

O -1.14055900 6.65105400 -0.58019900

O -0.88350000 6.00247800 -2.70053800

C 13.60567300 2.42732900 -4.20176700

C 12.62133300 2.79097200 -3.30462600

C 11.51577400 1.92775800 -3.08729800

C 11.42900700 0.69299000 -3.80713500

C 12.45041300 0.34449400 -4.72327800

C 13.52031400 1.19850200 -4.91345300

H 14.45529900 3.08051900 -4.37016100

H 12.66004200 3.71972500 -2.74713900

H 12.35849800 -0.59592000 -5.25407900

H 14.30635500 0.93518300 -5.61332100

C 9.43392200 0.20649400 -2.73889900

C 9.52462600 1.42960300 -2.00048900

H 8.58464600 -0.45197900 -2.61712900

N 10.53661300 2.28468000 -2.19744300

N 10.35239700 -0.14851700 -3.62590800

C 8.51662300 1.91191900 -1.05714400

H 8.58341100 2.97607100 -0.84971700

N 7.53640500 1.29558700 -0.47609800

C 8.07291700 -1.01414900 -0.10840000

O 9.30821000 -0.80593000 0.00775000

N 7.15348400 -0.03893600 -0.45606800

C 7.48183300 -2.36598400 0.12980900

C 6.15481200 -2.68205200 -0.21532800

C 8.30569600 -3.34900400 0.70541100

C 5.64816100 -3.95700000 0.03132100

H 5.53460700 -1.93302000 -0.69203100

C 7.81012000 -4.62575900 0.96006700

H 9.33012000 -3.08978000 0.94353500

C 6.48305900 -4.90882900 0.62392300

H 4.63321800 -4.22623000 -0.22938900

H 8.42391900 -5.39588100 1.40782400

N 5.94899600 -6.24532700 0.89974200

O 4.73871400 -6.47475100 0.60998100

O 6.72197100 -7.10092000 1.41904000

Ni 5.79368500 1.06997800 0.53506600

**TD –DFT calculation:**

| System | Experimental Wave length (nm) | Theoretical Wave length (nm) | Oscillator strength | Contribution orbitals |
| --- | --- | --- | --- | --- |
| Ni-1b complex | 442 | 431.6  443.3  454.2  454.6 | 0.053  0.072  0.002  0.004 | H-2(B)->L+1(B) (21%)  H-2(B)->L+1(B) (16%), H-1(B)->L+1(B) (16%)  H(A)->L+3(A) (53%)  H-1(A)->L(A) (62%) |
| 1b | 275 | 270.4  284.685395604 | 0.2335  0.1322 | H-4->L+1 (29%), H->L+2 (35%)  H-10->LUMO (47%) |
|  | 340 | 336.5 | 0.2474 | H->L+1 (84%) |
|  | 370 | 378.1 | 0.194 | H->L (91%) |

10. References:

1.(a) S. Goswami, S. Chakraborty, S. Paul, S. Halder, A. C. Maity, Tetrahedron Letters, 2013, 54, 5075.

(b)J. Cosier, A. M. Glazer. A nitrogen-gas-stream cryostat for general X-ray diffraction studies. J. Appl. Cryst.1986, **19**, 105–107.

2.Bruker. APEX2, SAINT and SADABS. Bruker AXS Inc.: Madison, WI, USA, 2009.

3. G.M. Sheldrick, A short history of SHELX. Acta Cryst. 2008, A64, 112-122.

4. M. J. Frisch, G. W. Trucks, H. B. Schlegel, G. E. Scuseria, M. A. Robb, J. R. Cheeseman, J. A Montgomery, T. Vreven, K. N. Kudin, J. C. Burant, J. M. Millam, S. S. Iyengar, J. Tomasi, V. Barone, B. Mennucci, M. Cossi, G. Scalmani, N. Rega, G. A. Petersson, H. Nakatsuji, M. Hada, M. Ehara, K. Toyota, R. Fukuda, J. Hasegawa, M. Ishida, T. Nakajima, Y. Honda, O. Kitao, H. Nakai, M. Klene, X. Li, J. E. Knox, H. P. Hratchian, J. B. Cross, V. Bakken, C. Adamo, J. Jaramillo, R. Gomperts, R. E. Stratmann, O. Yazyev, A. J. Austin, R. Cammi, C. Pomelli, J. W. Ochterski, P. Y. Ayala, K. Morokuma, G. A. Voth, P. Salvador, J. J. Dannenberg, V. G. Zakrzewski, S. Dapprich, A. D. Daniels, M. C. Strain, Farkas, O., Malick, D. K., A. D. Rabuck, K. Raghavachari, J. B. Foresman, J. V. Ortiz, Q. A. Cui, G. Baboul, S. Clifford, J. Cioslowski, B. B. Stefanov, G. Liu, A. Liashenko, P. Piskorz, I. Komaromi, R. L. Martin, D. J. Fox, T. Keith, M. A. Al-Laham,   C. Y. Peng, A. Nanayakkara, M. Challacombe, P. M. W. Gill, B. Johnson, W. Chen, M. W. Wong, C. Gonzalez, J. A. Pople, *GAUSSIAN 03 (Revision B.04)*; Gaussian, Inc.: Pittsburgh, PA, **2003**.

5. Becke, D. *J. Chem. Phys.* 1993, **98**, 5648

6. G. A. Petersson, M. A. Al-Laham, *J. Chem. Phys.* 1991, **94**, 6081

7. P. J. Hay, W. R. Wadt. *J. Chem. Phys.* 1985, **82**, 299. (b) P. J. Hay, W. R. Wadt. *J. Chem. Phys.* 1985, **82**, 270.
